# Editorials

**Published:** 1912-02

**Authors:** 


### EDITORIALS

The Business Office of the *Journal-Record* is 1 1-2 to 5 1-2 South Broad Street.
The Editorial Office is 1014-15 Century Building.
Address all Business Communications to *Journal-Record of Medicine*, 1 1-2 to 5 1-2 South Broad Street.
Make remittances payable *to The Journal-Record of Medicine.*

On matters pertaining to the Editorial and Original Communications, address Edgar G. Ballenger, M. D., Atlanta, Ga.
Reprints of Original Articles will be furnished at cost price. Requests for the same should always be made in THE MANUSCRIPT.

We will present, postpaid, on request, to each contributor of an original article, twenty (20) marked copies of *The Journal-Record of Medicine* containing such article.

### THE CHATTAHOOCHEE VALLEY MEDICAL AND SURGICAL ASSOCIATION.

The eleventh semi-annual session of the Chattahoochee Valley Medical Association was held at La Grange, Ga., January 16-17, 1912. This meeting was well up to the usual standard of these gatherings which have obtained a well-merited reputation for the cordial feeling among the members and for the scientific character of the papers read.

It is with pleasure that we publish in this number some of the papers presented at the La Grange meeting.

The next meeting is to be held in Atlanta and every effort will be made to make this a "banner meeting," as many of the members live in Atlanta and nearby territory. Essayists are urged to begin now the preparation of their papers so that they will bear the ear-marks of mature thought and work, and thus raise higher and higher the standard of the meetings. Dr. George M. Niles, Atlanta, was chosen president for the coming year.

### RESOLUTIONS OF RESPECT.

Chattahoochee Valley Medical and Surgical Association, La Grange, Ga., Jan. 17th, 1912.

Whereas, it has pleased Almighty God to remove from earth our highly esteemed brother and co-worker, Dr. A. H. Read, and

Whereas, we deeply deplore his death, and desire to place upon the permanent records of our Association some evidence and memorial of the high esteem in which our deceased brother was held by the members of our Association,

Therefore, be it resolved by the Chattahoochee Valley Medical and Surgical Associat'on, that in the death of Dr. Read, the Association has lost a useful and enthusiastic member; the medical profession an honored and faithful representative; the State a good citizen; his family a devoted father.

Resolved further, that we tender to the bereaved family our heartfelt sympathy.

Resolved further, that these resolutions be spread upon the minutes of this Association, that a copy thereof be sent to the family of the deceased, and that they be furnished to the Journal-Record of Medicine for publication.

T. H. HARALSON, M. D.,
O. V. LANGLEY, M. D.,
GEO. H. COOPER, M. D.,
Committee.

